# Epidemiology of Japanese Encephalitis in the Philippines: A Systematic Review

**DOI:** 10.1371/journal.pntd.0003630

**Published:** 2015-03-20

**Authors:** Anna Lena Lopez, Josephine G. Aldaba, Vito G. Roque, Amado O. Tandoc, Ava Kristy Sy, Fe Esperanza Espino, Maricel DeQuiroz-Castro, Youngmee Jee, Maria Joyce Ducusin, Kimberley K. Fox

**Affiliations:** 1 University of the Philippines Manila—National Institutes of Health, Institute of Child Health and Human Development, Manila, Philippines; 2 Epidemiology Bureau, Department of Health, Manila, Philippines; 3 Department of Virology, Research Institute for Tropical Medicine, Manila, Philippines; 4 Department of Parasitology, Research Institute for Tropical Medicine, Manila, Philippines; 5 Office of the World Health Organization Representative in the Philippines, Manila, Philippines; 6 Division of Communicable Diseases, World Health Organization Regional Office of the Western Pacific, Manila, Philippines; 7 Disease Prevention and Control Bureau, Department of Health, Manila, Philippines; U.S. Naval Medical Research Unit No. 2, INDONESIA

## Abstract

**Background:**

Japanese encephalitis virus (JEV) is an important cause of encephalitis in most of Asia, with high case fatality rates and often significant neurologic sequelae among survivors. The epidemiology of JE in the Philippines is not well defined. To support consideration of JE vaccine for introduction into the national schedule in the Philippines, we conducted a systematic literature review and summarized JE surveillance data from 2011 to 2014.

**Methods:**

We conducted searches on Japanese encephalitis and the Philippines in four databases and one library. Data from acute encephalitis syndrome (AES) and JE surveillance and from the national reference laboratory from January 2011 to March 2014 were tabulated and mapped.

**Results:**

We identified 29 published reports and presentations on JE in the Philippines, including 5 serologic surveys, 18 reports of clinical cases, and 8 animal studies (including two with both clinical cases and animal data). The 18 clinical studies reported 257 cases of laboratory-confirmed JE from 1972 to 2013. JE virus (JEV) was the causative agent in 7% to 18% of cases of clinical meningitis and encephalitis combined, and 16% to 40% of clinical encephalitis cases. JE predominantly affected children under 15 years of age and 6% to 7% of cases resulted in death. Surveillance data from January 2011 to March 2014 identified 73 (15%) laboratory-confirmed JE cases out of 497 cases tested.

**Summary:**

This comprehensive review demonstrates the endemicity and extensive geographic range of JE in the Philippines, and supports the use of JE vaccine in the country. Continued and improved surveillance with laboratory confirmation is needed to systematically quantify the burden of JE, to provide information that can guide prioritization of high risk areas in the country and determination of appropriate age and schedule of vaccine introduction, and to measure the impact of preventive measures including immunization against this important public health threat.

## Author Summary

Japanese encephalitis virus (JEV) is an important cause of neurologic infections in Asia, resulting in substantial disability and deaths. Although believed to be endemic in the Philippines, little is known of the epidemiology and geographic distribution of this disease in the country. We reviewed data from clinical studies, prevalence surveys and animal studies since the 1950s. Based on this review, JEV is an important cause of encephalitis and febrile illness in all three major island groups of the country. The majority of cases were seen in children younger than 15 years and males were more often affected than females. The national laboratory initiated testing of referred cases in 2009 and surveillance for acute encephalitis syndrome (AES) with laboratory confirmation of a subset of cases was established in 2011. From 2011 to 2014, there were 1,032 cases of suspected JE. Of 497 cases with specimens tested, 73 (15%) had laboratory-confirmed JE. Our findings confirm that JE has an extensive geographic distribution in the Philippines. These findings support the introduction of JE vaccine into the country’s routine immunization program.

## Introduction

Japanese encephalitis (JE) is a vector-borne disease that is endemic in most of Asia. Worldwide, it is estimated that around 68,000 cases occur annually, 40,000 in the Western Pacific Region alone. Most of these cases in endemic countries occur among children under 15 years of age, as adults are often already immune to the disease. JE is a significant public health threat, with case fatality rates of up to 30% and long-term neuropsychological sequelae in 30–50% of its survivors [1]. Because of the absence of treatment for JE and recent expansion of the geographic range of the disease, the World Health Organization (WHO) has recognized the exigency of improved surveillance for JE and recommended the integration of JE vaccine into routine immunization programmes wherever JE constitutes a public health problem. Vaccination is considered the single, most important control measure for JE [2].

Japanese encephalitis virus (JEV) circulation in the Philippines was first suggested when antibodies to JEV were identified in Philippine horses in 1943 [3]. Since then, JEV has been identified as a cause of encephalitis in humans in the Philippines and the country is believed to be endemic for the disease. However, the epidemiology of JE in the country has not been well defined. To assist the Government of the Philippines in its deliberations on the potential inclusion of JE vaccine in its routine immunization programme, we conducted a systematic literature review on the epidemiology of JE in the country. We also collated all available data from the country’s JE surveillance and laboratory referral systems.

## Methods

### Ethics

This study used published literature, presentations and disease surveillance data collected through existing, routine public health surveillance activities; no specific research approvals were required.

### The country

The Philippines has an estimated population of 100 million [4] and is an archipelago of approximately 7,107 islands located in the western Pacific Ocean in southeastern Asia. Geographically, the country is divided into 3 groups of islands: Luzon in the north, Visayas in the central region and Mindanao in the south. Administratively, the country is further subdivided into 17 regions encompassing the national capital region and 81 provinces. The country has two seasons, the rainy season from June to November and the dry season from December to May [5,6].

### Systematic review

We conducted searches on Pubmed and ProMed using the search terms “Philippines” and “Japanese encephalitis”. Further searches were conducted in the Philippines Index Medicus, the Health Research and Development Information Network (HERDIN) which is the national health research repository of the Philippines and the Department of Health (DOH) National Epidemiology Center (NEC) library, which has a repository of outbreak investigations. We also retrieved the articles in the reference lists of papers found in our searches. Presentations by JE researchers to WHO were identified and obtained. Data from these researches were verified to ensure that these were not included in other published literature.

Two authors (ALL and JGA) reviewed the list of articles and presentations separately. The number of articles and presentations reporting on serologic surveys, clinical infections and animal data were tabulated separately to ensure that all of the information from the articles and presentations was obtained. Inconsistencies between the tabulations were discussed and resolved between the two reviewers.

### Surveillance for Japanese encephalitis

As part of the Philippine Integrated Disease Surveillance and Response, surveillance for acute encephalitis syndrome (AES), as a proxy for JE, was established in 2008. This surveillance consisted of a simple line listing of AES, or suspected JE, cases without laboratory confirmation. In addition, more detailed case-based sentinel surveillance for JE with laboratory confirmation was initiated in 2012 in four hospitals: Northern Mindanao Medical Center, Philippine Children’s Medical Center, San Lazaro Hospital and Western Visayas Medical Center. A fifth hospital, Bicol Medical Center, was added in 2013. These hospitals were selected based on several criteria: geographically situated to represent the major regions of the country, routinely perform lumbar puncture and have the ability to transport CSF and serum specimens to the national laboratory for testing. Case definitions were based on the WHO surveillance standards for JE (Box 1).

**Box 1. Case definitions used in Japanese encephalitis syndrome surveillance**

Acute encephalitis syndrome (AES)

- Defined as a person of any age, at any time of year with the acute onset of fever and at least one of:
  - Change in mental status (including symptoms such as confusion, disorientation, coma, or inability to talk)
  - New onset of seizures (excluding simple febrile seizures*).
- Other early clinical findings may include an increase in irritability, somnolence or abnormal behavior greater than that seen with usual febrile illness.

AES (suspected JE) case

- Defined as a case that meets the clinical case definition for AES
  Laboratory-confirmed JE
- An AES case with JEV-specific IgM antibody in a sample of CSF or serum detected by IgM-capture ELISA

*A simple febrile seizure is defined as a seizure that occurs in a child aged 6 months to less than 6 years old, whose only finding is fever and a single generalized convulsion lasting less than 15 minutes, and who recovers consciousness within 60 minutes of the seizure.

After WHO established a JE laboratory network in the Western Pacific Region in 2009, the Philippines DOH designated the Research Institute for Tropical Medicine (RITM) as the national JE laboratory. Clinicians referred specimens from suspected JE cases to RITM for testing. Specimens from sentinel surveillance and clinician referral were assayed for JE-specific IgM following WHO guidelines for case confirmation (Box 1).

Data on suspected JE cases from the line list, and suspected and confirmed JE cases from the sentinel surveillance and clinician referral systems, were tabulated and mapped.

## Results

**Fig 1:**
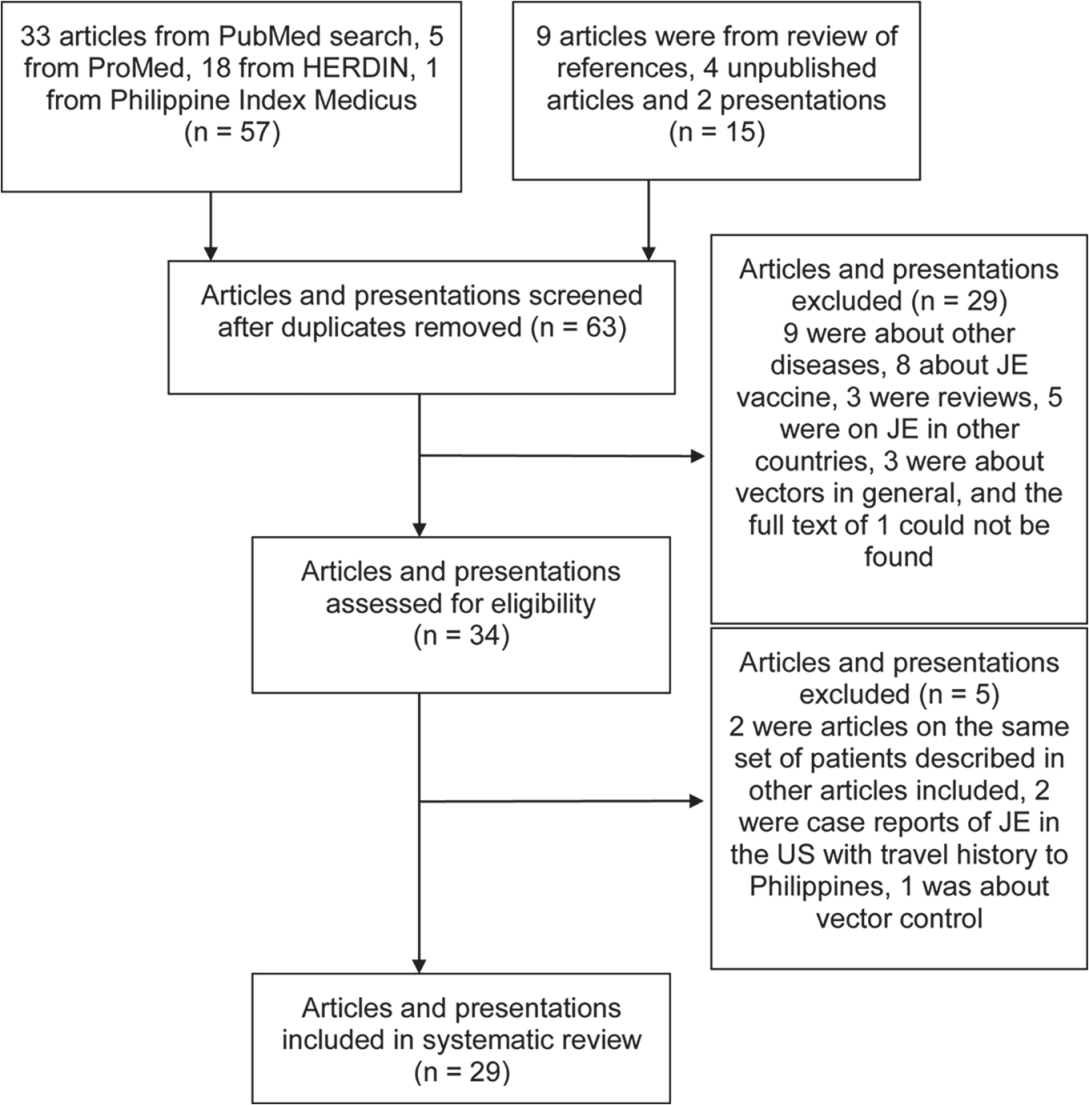
Assessment of studies for inclusion in the systematic review.

We identified 29 articles and presentations on JE in the Philippines (Fig. 1). Five articles published from 1958 to 1993 reported on JE serologic surveys conducted in various areas of the country (Table 1). The first serologic survey in JE in the Philippines was conducted by Hammon, et al, using the suckling mouse neutralization test (NT) [3]. In 1964, Basaca-Sevilla and Halstead included nine serological studies conducted in the Philippines that identified JE antibodies using HI, CF or NT in their review, suggesting the circulation of JEV in the country [7].

**Table 1:** Serologic surveys for Japanese encephalitis in the Philippines, 1958–1993.

	Location	Subjects tested	Age groups (n)	% JE positive among those tested	Confirmatory test
Hammon, et al, 1958 [3]	Pampanga	Indigenous residents in the Jungle	6 mos to 4 yrs (n = 18)	39%	NT
			5 to 9 years (n = 23)	78%	
			10 to 14 years (n = 6)	83%	
			15+ years (n = 77)	99%	
		Village near military base	10 to 14 years (n = 52)	23%	
			15+ years (n = 51)	49%	
	Manila	Attendees in out-patient clinic excluding suspected polio and encephalitis cases	6 mos to 4 yrs (n = 32)	3%	
			5 to 9 years (n = 23)	17%	
Macasaet, et al, 1970 [38]	Negros Oriental	School children	6 to 12 years (n = 1,008)	60%	HI
Arambulo, 1974 [39]	Negros Oriental	Adults and children	Not stated	92%	Not stated
Cross, et al, 1977 [40]	Samar	Residents of 8 communities	All ages (n = 1201)	78%	HI
Radda, et al, 1993 [41]	Oriental Mindoro	Outpatient clinic attendees for different disorders, including fever	All ages (n = 129)	29%	HI

NT—Neutralization tests; HI—Hemagglutination inhibition

Clinical JE was reported in 18 articles and presentations. The first serologically confirmed case of JE acquired in the Philippines was reported in 1956 in an American soldier stationed in the country [8]. Subsequent cases of possible JE were detected during investigations of clinically suspected dengue fever cases. In an investigation by Macasaet et al in Negros Oriental in the Visayas, samples from 10 patients with clinical dengue and their contacts were examined for antibodies to various arboviruses including JEV. Antibodies to JEV and dengue were detected using HI assay and one case had a significant rise in titer to JEV but not to dengue viruses, suggesting acute infection with JEV [9].

In 1972, Venzon et al reported the first systematic testing of CSF and serum specimens from encephalitis cases, identifying JEV as an important cause of encephalitis in the Philippines [10]. Table 2 summarizes studies of clinical JE in the Philippines from 1972 to 2013. Overall, these reports include 257 laboratory-confirmed JE cases. Among studies that tested suspected meningitis and encephalitis cases combined, 7% to 18% were JE-positive. Among studies that tested clinical encephalitis cases, 16% to 40% were JE-positive. A large majority of cases were among children under 15 years old, and the male-to-female ratio of cases ranged from 1.1 to 3.0.

**Table 2:** Summary of reports on clinical Japanese encephalitis (JE) in the Philippines, 1972–2013.

	Details	No. of cases tested and type of specimen	No. (%) confirmed JE	Age and sex distribution of JE cases	Length of hospital stay and outcome of JE cases	Peak Months
Venzon et al, 1972 [10]	Clinically diagnosed meningitis and encephalitis cases in 8 Metro Manila hospitals in 1969–1971; used HI assay for confirmation	114 cases with paired sera	20 (17%)	Not stated	Not stated	Not stated
Chan and Samaniego, 1983 [26]	Clinically diagnosed viral encephalitis cases from Metro Manila, Bacolod and Cebu in 1979–1980; used HI assay for confirmation	38 cases, 16 with paired sera and 22 with single serum samples	6 (16%)	2 cases 2 years old, others not stated	Not stated	Not stated
Barzaga, 1989^a^ [12]	Prospective surveillance for JE in San Lazaro Hospital in Manila and in Cabanatuan, Nueva Ecija in 1985; used IgM capture ELISA for confirmation	129 cases CSF and sera in Manila	22 (17%)	88% of cases among 1 to 15 years old	5 deaths out of 69 cases from Manila and Cabanatuan with information (CFR: 7.2%)	August-September
		ND for Cabanatuan	54 confirmed JE cases	85% cases <15 years old; M:F ratio 1.1		Not stated
San Luis, et al, 1990^b^ [11]	Prospective surveillance for JE in San Lazaro Hospital in Manila	ND, CSF and sera	52^c^ confirmed JE cases	90% of cases <15 years old (among 51 cases with information); ND	At 3 months of follow-up of 51 JE cases, there were 3 deaths (CFR, 5.9%). Average hospital stay was 30 days (range 20–46)	Not stated
Mayo, 1998 [13]	Series of cases of JE admitted in Cotabato Regional Hospital in June 1989–May 1990; Used IgM capture ELISA for confirmation	ND	14 cases	All cases 3 to 14 years old; M:F ratio 1.8	71% of JE cases stayed for 11–20 days in hospital, 1 death (CFR 7%);	February, June
Inoue, 2002[24]	Clinically diagnosed meningitis and encephalitis cases from patients in St. Luke's Hospital and other hospitals in Metro Manila, Luzon and Visayas; used IgM capture ELISA for confirmation	203 cases with CSF and/or sera	15 (7%)	Not stated	Not stated	Not stated
Natividad, et al, 2006 [25,42]	Clinically diagnosed meningitis and encephalitis cases from patients in Metro Manila, Luzon and Visayas; Used IgM capture ELISA for confirmation	614 cases with CSF samples	72 (12%)	Of 48 cases with age data, 73% <17 years old; M:F ratio 2.7	Not stated	Not stated
Latorre-Mendoza, 2007 [15]	Series of JE cases admitted in Philippine General Hospital in 2006; used IgM capture ELISA for confirmation	ND, CSF samples	4 cases in 2006	All cases 3 to 11 years old; M:F ratio 3.0	All cases had neurologic sequelae on discharge	Not stated
Alera, 2013 [14]	Cases with acute encephalitis syndrome ≥2 years old in San Lazaro Hospital from September 2005 to December 2006; used IgM capture ELISA for confirmation	15 cases with CSF and sera	6 (40%)	All cases 3 to 14 years old; M:F ratio 2.0	Average length of stay 22 days (range 11 to 31), 50% had neurologic sequelae on discharge	July
Espino, 2013 [16]	Clinically diagnosed meningitis and encephalitis cases from patients <18 years old in hospitals in Bicol, Bulacan, Iloilo, Quezon City and Tarlac; used IgM capture ELISA for confirmation	251 cases	44 (18%)	All cases <15 years old, 64% 2–9 years old; M:F ratio 1.5	On discharge, 17% had neurologic sequelae	Not stated

^a^ This article presents initial results of the surveillance conducted by the US Naval Medical Research Unit No. 2 [43]; complete results of the surveillance were not available.

^b^ This article presents the neurologic outcome of patients described by Barzaga [12].

^c^ This figure is not included in the overall total number of JE cases from articles and presentations because of substantial overlap with cases reported by Barzaga [12]

ND, No data presented

CFR, case fatality ratio

Only one study reported outcomes at least three months after discharge. Among 48 JE survivors, 65% became functionally independent although half of them had neurologic deficits, 17% remained functionally dependent with neurologic sequelae and the rest were lost to follow-up [11]. Five studies reported on the close proximity of JE cases to rice fields [12–16] and three reported on the practice of swine propagation near the residence of JE cases [14–16]. In one study, a significant positive association was seen with both proximity to rice fields and contact with pigs among JE cases but not with other cases of CNS infections [16].

**Fig 2:**
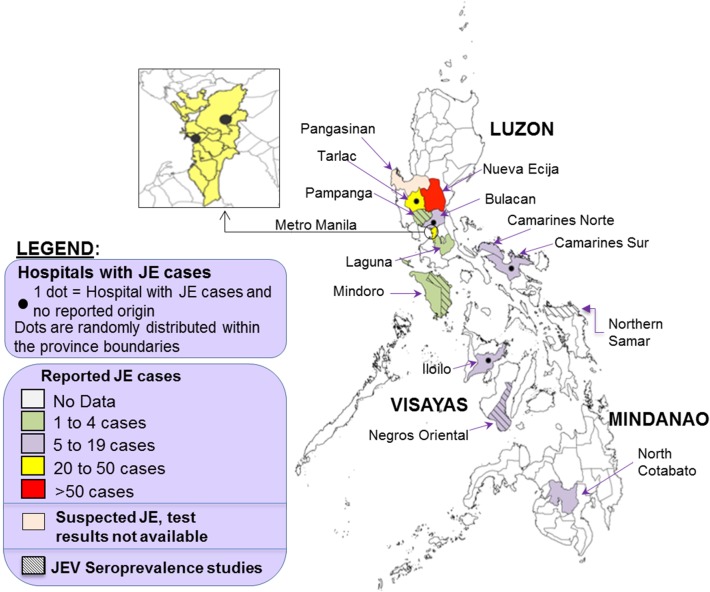
Geographic distribution of reported JE cases, suspected JE cases and seroprevalence surveys in the Philippines, by province. Data from published reports and presentations, 1958–2013. Some reports did not specify origin of cases; in these cases, location of the hospital was mapped (black dots). The number of reported JE cases may have been biased by factors other than the incidence of JE. The preference for certain study sites and duration of studies may have unduly increased the number of cases in some provinces. There were additional cases with no detailed residence information: 28 cases in Luzon and 7 cases in Visayas. A seroprevalence survey was also conducted in Manila, but obscured by overlapping black dot for hospital.

The first report of a JE outbreak was from Nueva Ecija in central Luzon in 1982 [17,18]. Subsequently, one suspected outbreak in Cotabato [19] and a series of cases in Pangasinan [20] were investigated but laboratory test results are unavailable. Fig. 2 shows the geographic distribution of reported confirmed JE cases, suspected JE cases and seroprevalence surveys from 1958 to 2013. JEV activity was identified in the Bicol region, in Metro Manila and in 10 additional provinces. (S1 Table)

**Fig 3:**
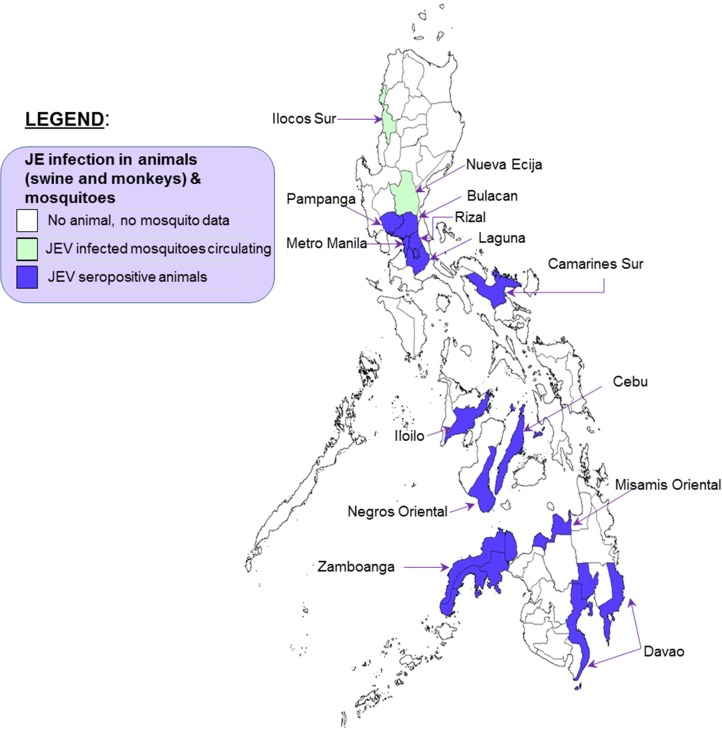
Geographic distribution of animal and mosquito studies in the Philippines, 1958–2012.

Since the first identification of antibodies to JEV in horses in 1943 [3], eight articles reported on JEV antibodies in swine and other animals in the Philippines (Table 3). Overall, 18% to 46% of pigs and 2.5% to 35% of monkeys had antibodies to JEV. Through serial follow-up of pigs, JEV transmission was found to occur primarily during irrigation periods or during the rainy season. The first isolation of JEV in the Philippines was in 1977 from pools of *Culex tritaeniorhynchus* and *C*. *vishnui* mosquitoes from Tagudin, Ilocos Sur in northern Luzon. These two mosquito species accounted for 74% of all mosquitoes collected. No JEV was isolated from other mosquitoes [21]. In 1980, JEV was isolated from *C*. *tritaeniorhynchus*, *C*. *bitaeniorhynchus* and *Anopheles annularis* mosquitoes in Nueva Ecija in Central Luzon [22]. Fig. 3 shows the geographic distribution of JEV circulation documented in animals and mosquitoes in Metro Manila and 13 provinces. (S1 Table)

**Table 3:** Japanese encephalitis studies in animals in the Philippines, 1966–2005.

	Details	Findings
Campos, 1966 [44]	109 native pigs from Luzon (Manila), Visayas (Iloilo, Dumaguete, Cebu) and Mindanao (Davao, Misamis, Cagayan de Oro); Sera obtained prior to slaughter	24% seropositive by HI^a^ (JE antibodies were identified among pigs from all locations)
Macasaet, 1970 [9]	256 animals including 112 bats, 23 wild birds, 34 pigs in Negros Oriental	80% of animals, including 91% of wild birds and 18% of pigs positive for JE antibodies by HI test
Arambulo, 1974 [39]	21 pigs in Negros Oriental	21% positive for JE antibodies by HI test
Shultz, 1993 [45]	Seronegative pigs followed up for seroconversion for 2 years to assess JEV activity in Nueva Ecija	Periods of JEV activity identified during irrigation period (February to March) and rainy season (June to September)
Natividad, 2005 [46]	121 healthy pigs from Luzon (Olongapo), Visayas (Iloilo), and Mindanao (Zamboanga)	5.8% of pigs were positive for IgM, while 46% of 117 sera were positive for IgG to JEV using IgM-capture and IgG ELISA, respectively
Guazon, 2012 [47]	43 pigs from slaughterhouse in Camarines Sur	12 (28%) were positive for antibodies to JEV by HI assay
Bain, 1988 [48]	120 serum samples from monkeys, *Macaca mulatta* and *M*. *fascicularis* in farms located in Iloilo, Laguna and Metro Manila	2.5% of monkeys from Laguna and Iloilo were positive for antibodies to JEV by HI assay
Inoue, 2003 [49]	54 monkeys (*M*. *fascicularis*) in a monkey farm in Rizal; 44 of the monkeys came from Zamboanga	35% of all monkeys positive for IgM to JEV using IgM-capture ELISA

^a^HI—Haemagglutination inhibition

### Surveillance and referral testing for Japanese encephalitis

From January 2011 to March 2014, 1,032 suspected JE cases were reported. Of 497 cases with specimens, 73 (15%) had laboratory-confirmed JE. Annually, the percentage of tested cases that were positive for JE IgM ranged from 11% to 25%. Clinician referral accounted for a larger proportion of these specimens (67%) than did surveillance (33%). During calendar years 2011–2013, 60 to 69 hospitals reported suspected JE cases annually, out of 171 that ever reported. A subset of 29 to 58 hospitals each year (out of 85 hospitals) submitted specimens for testing. Table 4 shows the yearly distribution of suspected JE cases, cases tested and confirmed JE cases from surveillance and the hospital referral systems.

**Table 4:** Yearly distribution of suspected and confirmed JE cases from surveillance and clinician referral testing, January 2011 to March 2014.

	No. of suspected JE cases*	No. of cases tested for JE	No. (%) confirmed JE
2011	199	64	16 (25%)
2012	352	129	24 (19%)
2013	392	237	25 (11%)
2014 (Jan-Mar)	89	67	8 (12%)
2011–2014	1032	497	73 (15%)

*Includes surveillance cases with and without specimens, and clinician referral cases.

**Fig 4:**
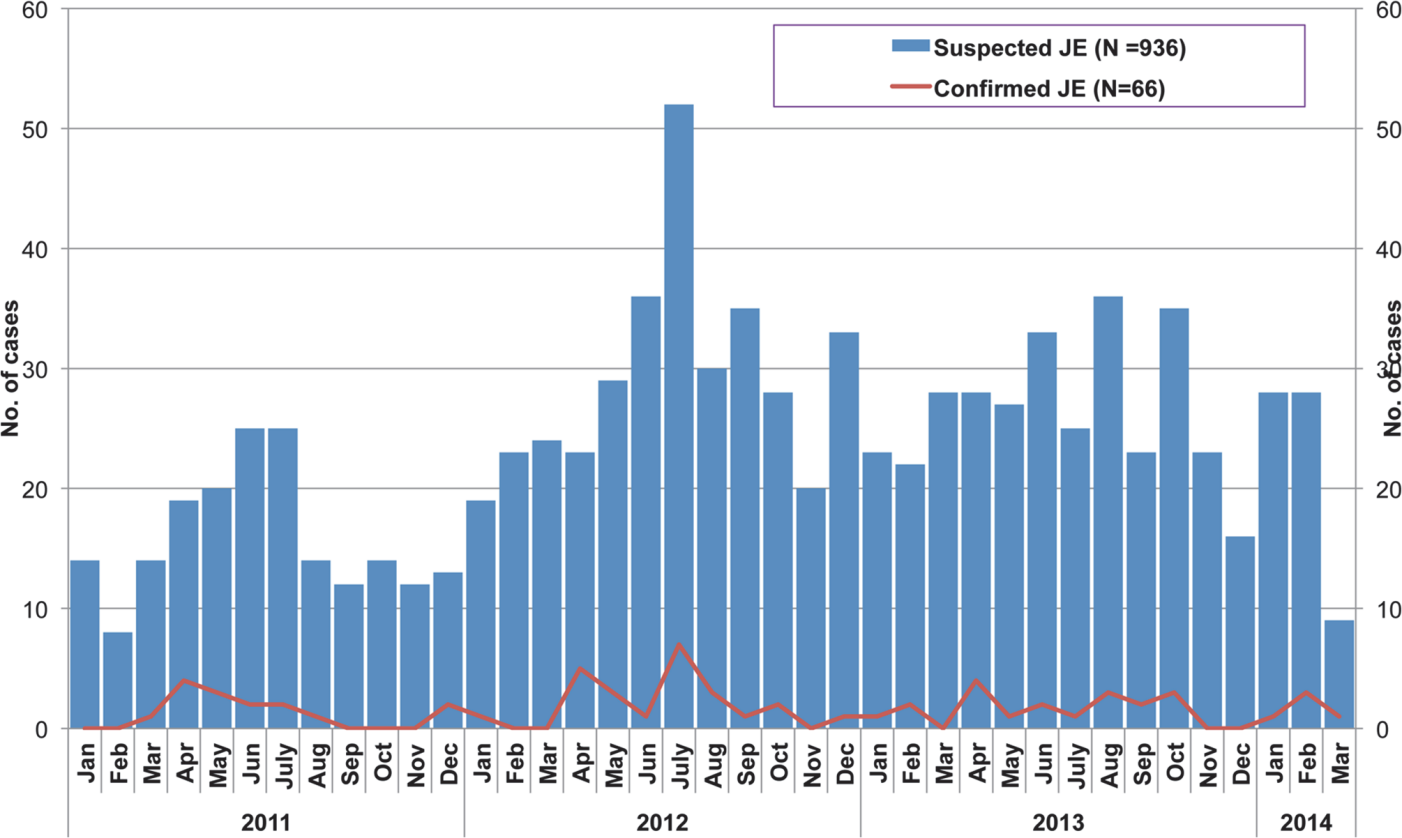
Monthly distribution of suspected and confirmed JE cases, January 2011 to March 2014. Suspected JE cases include line-listed cases without laboratory confirmation, sentinel surveillance cases and clinician referral cases. Of 1032 suspected JE cases, 497 (48%) underwent laboratory testing for JE.

In the surveillance data, June and July were the months with the highest numbers of suspected JE cases and November was the month with the fewest suspected JE cases (Fig. 4). Among the 70 confirmed JE cases with information on the month of illness, the highest percentage occurred in April (19%), followed by July (14%), August (11%) and May (10%). Confirmed JE cases occurred in all months except November.

**Fig 5:**
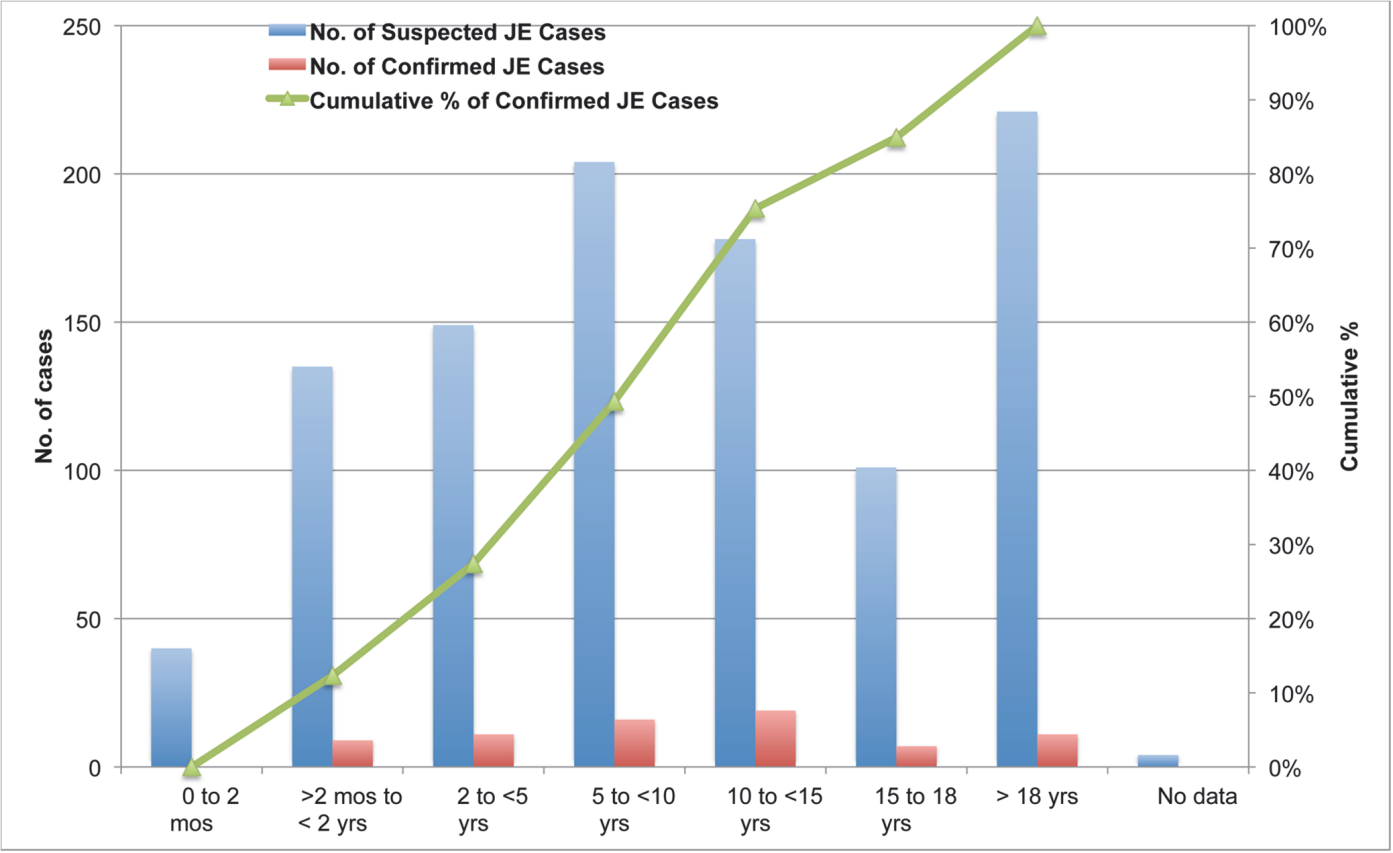
Age distribution of suspected (n = 1032) and confirmed (n = 73) JE cases and cumulative percentage of confirmed JE cases, January 2011 to March 2014. Suspected JE cases include simple line listing of AES cases without laboratory confirmation, case-based sentinel surveillance for JE with laboratory confirmation and clinician referred specimens for testing to RITM.

The majority of suspected JE cases were children: 68% were under 15 years old and 78% were under 19 years old. Among confirmed JE cases, 75% were children under 15 years of age and 85% were under 19 years old (Fig. 5).

**Fig 6:**
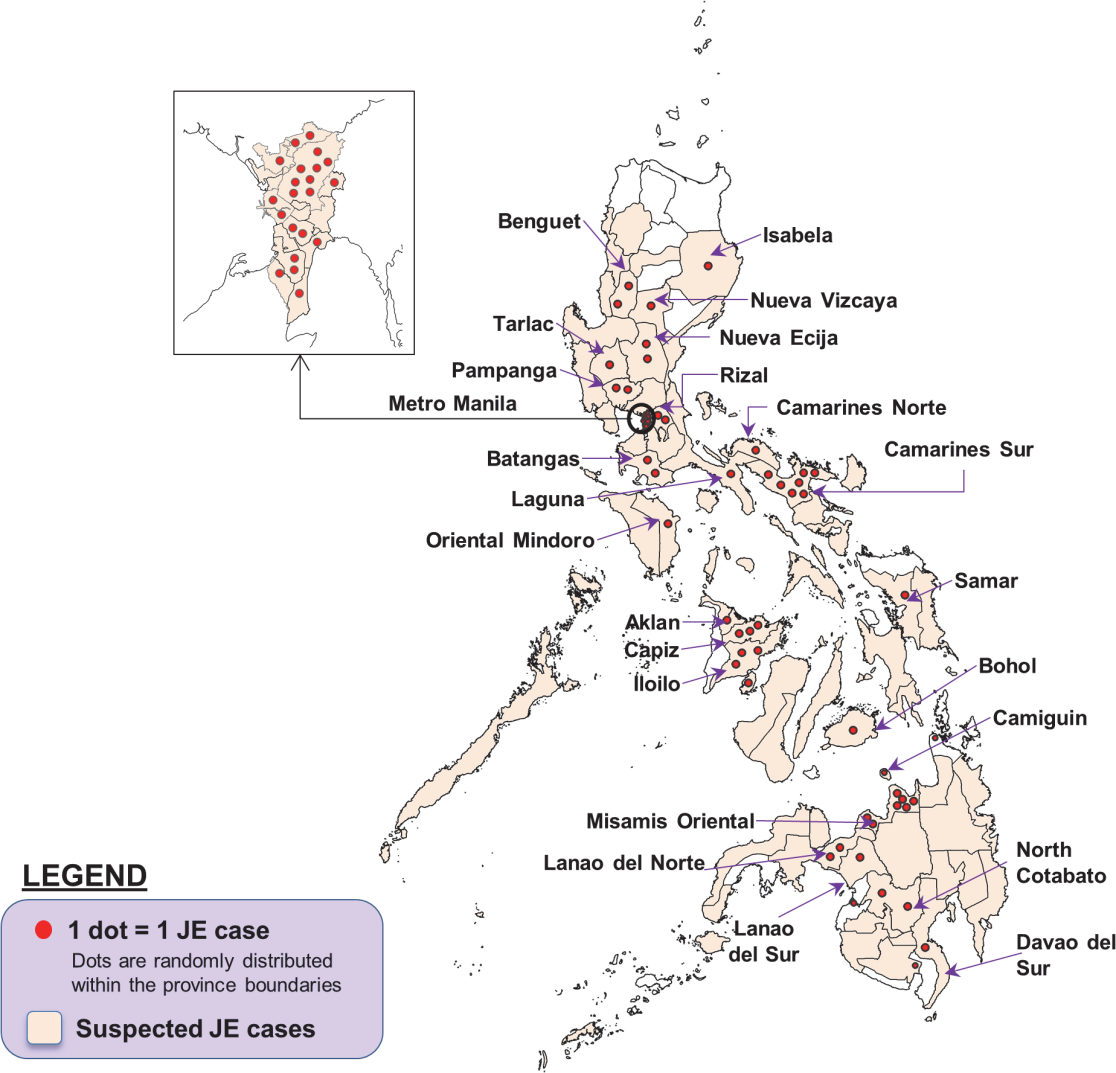
Geographic distribution of suspected and confirmed JE cases in the Philippines. Data from surveillance and referral testing, January 2011 to March 2014. There were additionally 21 confirmed JE cases out of 159 cases referred by hospitals in Metro Manila without available data on geographic origin.

Fig. 6 shows the geographic distribution of suspected and confirmed JE cases. Each region had at least one suspected JE case reported. Among the 81 provinces, there were 68 that reported suspected JE cases and 20 that reported confirmed JE cases. (S2 Table).

## Discussion

This systematic review of JE data confirms the endemicity of JE in the Philippines and its broad distribution across the country. Based on seroepidemiologic and clinical JE infection studies, animal and mosquito surveys, and surveillance data, JEV has been documented in Metro Manila and in 32 provinces located in all regions of the country and suspected JE cases have been reported in 68 of the 81 provinces and major cities of the country. Mapping of available data on JE in the country suggests that the disease is widespread.

Many pathogens other than JEV cause AES and the estimated incidence of AES is 6 per 100,000 population per year across all age groups [23]. Based on this incidence, about 6,000 AES cases are expected in the Philippines annually. However, surveillance and clinician referral data from January 2011- March 2014 identified only 1,032 cases over 39 months, or around 318 cases annually, 5% of the expected national number of AES cases. JE cases may have been similarly underrecognized or underdiagnosed if specimens were collected early in the disease and no follow-up sera were obtained. In the studies included in this review, JE was responsible for 7% to 18% of meningitis and encephalitis cases combined [10–12,24–26], and 16% to 40% of encephalitis cases [14]. If there are 6,000 annual AES cases then we estimate that a minimum of 10–15% of those are JE, or 600 to 900 JE cases annually in the country. During 39 months of surveillance and referral testing with specimens submitted by 85 hospitals, only 73 confirmed JE cases, around 2–4% of the expected national JE number, were identified. These calculations suggest that the lack of reported AES and JE cases in some areas may reflect limitations in surveillance rather than absence of disease transmission.

As in other Asian countries prior to incorporation of JE vaccine into the national immunization program, JE in the Philippines predominantly affects children under 15 years of age, causes substantial mortality [11–13], and results in significant neurologic sequelae in a majority of survivors [11,15,27]. In endemic areas, serological surveys suggest that most of the residents are infected by 15 years of age and immune to JE, accounting for the smaller number of cases found in adults. This pattern is similar to that seen in other endemic areas and consistent with the expected ratio of subclinical infection to clinical encephalitis of 250–500 to 1 [2]. As in other tropical countries, JE represents an ongoing year-round threat in the country [28,29]. Although children are predominantly affected, studies as well as surveillance data suggest that adults are still at risk with 15% of cases occurring in individuals older than 18 years.

The case fatality rate found in this review, 6–7%, is lower than the estimated rate of up to 30% [1]. Some of the cases may have been arrived severely ill and died before samples were obtained, or were taken home to die and thus were not counted, furthermore, cases that resulted in death without reaching a hospital were not measured and may lead to the underestimation of the true case fatality rate.

Our study has several limitations. First, we were unable to obtain some of the articles from the 1950s and 1960s, so we relied on review articles for some of the early data. We avoided duplication in reporting and included information from those with the most comprehensive data. Second, substantial cross-reactivity exists among arboviruses, particularly with HI and CF tests. Thus, some of the positive serologic tests, particularly before 1990, may have reflected infection with other arboviruses. However, for most of the clinical cases during that time period, studies required 4-fold rises in titers against JEV without increases in titers against other arboviruses including dengue virus, to classify cases as JE. Third, some of the presentations were not published. However, we felt that the information from these sources was important to include in the review. Fourth, although there are many studies, most were conducted in a small number of sites and metropolitan centers, thus the geographic range of data before the 2012 initiation of AES surveillance is limited. Fifth, laboratory confirmation was not available for some encephalitis outbreaks. We reported these as suspected JE cases. Lastly, the surveillance and referral testing data included cases from three sources: line-listed AES cases without laboratory testing, sentinel surveillance and clinician referral testing. It is therefore not surprising that only 48% of cases had specimens tested. Sentinel surveillance covers only five hospitals and is relatively new so may not be fully implemented yet. As the sentinel surveillance system matures, it is hoped that high and stable enrollment will provide a more accurate measure of JE prevalence among encephalitis cases in the sentinel locations, and a reliable source for monitoring trends in JE epidemiology in the country.

The continued risk for JE infection in the Philippines is also reflected in the reported cases among travellers to the country. A review of 55 JE cases from 1973–2008 among travellers from non-endemic regions revealed 5 (9%) were contracted in the Philippines. Among these 5 cases, 2 died of JE [30,31]. More recently, two additional cases were reported, one of which was fatal [32,33].

The conditions for JEV propagation are present in the Philippines. Although the Philippines has comparatively less land (4.35 million hectares in 2010) devoted to rice production than its neighbors, rice production tripled since the 1970s [34]. Swine production in the Philippines is the largest contributor to the country’s agriculture after rice production, with 71% of swine production dependent on backyard farm-raising [35]. In an ecological niche model, it was estimated that 46% of the land area of the Philippines had a >25% probability of the presence of the vector, *C*. *tritaeniorhynchus*. The model predicted that land areas with *C*. *tritaeniorhynchus* presence coincided with the areas where rice is cultivated [36]. Even if the *C*. *tritaeniorhynchus* is present in less than half of the land area, the rice field habitat of the mosquitoes may be located in close proximity to large populations, facilitating JEV transmission to humans.

This review documented JEV in all regions of the Philippines and found JEV to be an important cause of encephalitis in all studies of clinical disease, with epidemiologic characteristics comparable to those found in other endemic countries in Asia. The evidence suggests that JE constitutes a significant public health burden in all regions of the Philippines, and supports consideration of the inclusion of JE vaccine in the national immunization programme. A decision on vaccine introduction will consider this evidence of disease burden, cost and cost-effectiveness, and operational factors. The WHO recommends as the most effective immunization strategy a one-time campaign in a locally-defined target population followed by incorporation of the JE vaccine in the routine programme [2]. Based on our findings, a one-time campaign vaccinating children under 15 years of age followed by inclusion of JE vaccine in the routine programme for young children would be suitable to the disease epidemiology. Areas with larger numbers of known cases or higher proportions of encephalitis found to be JE could be prioritized if vaccine is introduced in a phased manner.

Continued and improved surveillance is needed to systematically quantify the burden of disease over time. The Government of the Philippines has developed plans to expand sentinel JE surveillance with laboratory confirmation to collect systematic data from all regions of the country and to provide a baseline for measuring vaccine impact. The WHO Regional Office for the Western Pacific provides technical support to countries to strengthen the WHO-designated national JE laboratory and surveillance capacity and to introduce JE vaccine [37].

## Supporting Information

S1 Checklist (DOCX)

S1 Table Confirmed and possible Japanese encephalitis (JE) cases, seroprevalence, animal and mosquito studies from 1958 to 2013. (DOCX)

S2 Table Suspected and confirmed JE cases in the Philippines from surveillance and referral testing, January 2011 to March 2014. (DOCX)
