# Supplementary material for: Epidemiology of Japanese Encephalitis in the Philippines: A Systematic Review
Source: PLoS Negl Trop Dis. 2015 Mar 20;9(3):e0003630. doi: 10.1371/journal.pntd.0003630 (PMC4367992; doi:10.1371/journal.pntd.0003630)
Supplement: S1 Table — (DOCX) [file pntd.0003630.s002.docx]

Supporting information

S1 Table. Confirmed and possible Japanese encephalitis (JE) cases, seroprevalence, animal and mosquito studies from 1958 to 2013

| Place | JE-confirmed cases | JE possible | Isolation of virus from Mosquitoes | Seroprevalence of JE in animals | References (Human studies) | References (Mosquitoes) | References (Animal studies) |
| --- | --- | --- | --- | --- | --- | --- | --- |
| Pampanga (areas around Clark Field Airbase) and Manila | 1 |  |  | Y | 3, 24 |  | 48 |
| Metro Manila | 37 |  |  | Y | 26, 17, 12, 24, 25 |  | 44, 47 |
| Ilocos Sur |  |  | Y |  |  | 21 |  |
| Iloilo |  |  |  | Y |  |  | 44, 46, 48 |
| Cebu |  |  |  | Y |  |  | 44 |
| Davao |  |  |  | Y |  |  | 44, 48 |
| Misamis |  |  |  | Y |  |  | 44 |
| Cagayan De Oro |  |  |  | Y |  |  | 44 |
| Negros Oriental | 6 |  |  | Y | 39, 38 |  | 38, 39 |
| North Samar |  |  |  |  | 40 |  |  |
| Nueva Ecija | 55 |  | Y | Y | 12, 42 | 22 | 44 |
| Rizal |  |  |  | Y |  |  | 49 |
| Zamboanga |  |  |  | Y |  |  | 49 |
| North Cotabato | 14 | Possible |  |  | 13, 19 |  |  |
| Pangasinan |  | Possible |  |  | 20 |  |  |
| Oriental Mindoro |  |  |  |  | 41 |  |  |
| Tarlac | 7 |  |  |  | 42 |  |  |
| Bulacan | 2 |  |  | Y | 24 |  | 49 |
| Laguna | 1 |  |  | Y | 24 |  | 48 |
| Bicol region (including Camarines Sur and Norte) | 3 |  |  | Y | 15 |  | 47 |
| Mindoro (both Oriental and Occ Mindoro) | 1 |  |  |  | 15 |  |  |
| Bicol Medical Center (BMC); Bulacan Medical Center; (BulMC) Philippine Children's Medical Center (PCMC); Tarlac Provincial Hospital (TPH); Western Visayas Medical Center WVMC) in Iloilo | BMC - 14; BulMC - 6; PCMC - 5; TPH - 14; WVMH - 5 |  |  |  | 16 |  |  |
| Not mapped |  |  |  |  |  |  |  |
| Luzon | 28 |  |  |  | 25 |  |  |
| Visayas | 7 |  |  |  | 25 |  |  |
