# Supplementary material for: Epidemiology of Japanese Encephalitis in the Philippines: A Systematic Review
Source: PLoS Negl Trop Dis. 2015 Mar 20;9(3):e0003630. doi: 10.1371/journal.pntd.0003630 (PMC4367992; doi:10.1371/journal.pntd.0003630)
Supplement: S2 Table — (DOCX) [file pntd.0003630.s003.docx]

Supporting information

S2: Table. Suspected and confirmed JE cases in the Philippines from surveillance and referral testing, January 2011 to March 2014

| Province/City | Suspected JE cases | Tested suspected cases | Total JE |
| --- | --- | --- | --- |
| National Capital Region |  |  |  |
| City of Las Piñas | 15 | 10 | 0 |
| City of Makati | 1 | 0 | 0 |
| City of Malabon | 4 | 3 | 0 |
| City of Mandaluyong | 2 | 1 | 0 |
| City of Manila | 85 | 8 | 0 |
| City of Marikina | 2 | 0 | 0 |
| City of Muntinlupa | 3 | 3 | 0 |
| City of Navotas | 0 | 0 | 0 |
| City of Parañaque | 5 | 2 | 0 |
| City of Pasig | 3 | 1 | 0 |
| City of San Juan | 1 | 0 | 0 |
| City of Valenzuela | 7 | 3 | 0 |
| Caloocan City | 17 | 2 | 1 |
| Pasay City | 2 | 2 | 0 |
| Pateros | 1 | 1 | 0 |
| Quezon City | 38 | 12 | 2 |
| Taguig City | 4 | 3 | 0 |
| NCR (no specific city) | 159 | 159 | 21 |
| Cordillera Administrative Region |  |  |  |
| Abra | 1 | 0 | 0 |
| Apayao | 0 | 0 | 0 |
| Benguet (excluding Baguio City) | 8 | 2 | 2 |
| Baguio City | 4 | 1 | 0 |
| Ifugao | 0 | 0 | 0 |
| Kalinga | 0 | 0 | 0 |
| Mountain Province | 1 | 0 | 0 |
| Region I – Ilocos Region |  |  |  |
| Ilocos Norte | 0 | 0 | 0 |
| Ilocos Sur | 1 | 0 | 0 |
| La Union | 1 | 0 | 0 |
| Pangasinan | 8 | 5 | 0 |
| Region II – Cagayan Valley |  |  |  |
| Batanes | 0 | 0 | 0 |
| Cagayan | 0 | 0 | 0 |
| Isabela | 12 | 2 | 1 |
| Nueva Vizcaya | 4 | 1 | 1 |
| Quirino | 0 | 0 | 0 |
| Region III – Central Luzon |  |  |  |
| Aurora | 1 | 0 | 0 |
| Bataan | 1 | 0 | 0 |
| Bulacan | 24 | 8 | 0 |
| Nueva Ecija | 38 | 11 | 2 |
| Pampanga | 12 | 5 | 2 |
| Tarlac | 27 | 5 | 1 |
| Zambales | 2 | 1 | 0 |
| Unknown | 2 | 2 | 1 |
| Region IVA – CALABARZON |  |  |  |
| Batangas | 20 | 8 | 2 |
| Cavite | 29 | 21 | 0 |
| Laguna | 20 | 12 | 0 |
| Quezon | 13 | 8 | 1 |
| Rizal | 24 | 15 | 2 |
| Unknown (admitted in Region IVA hospital) | 3 | 3 | 0 |
| Region IVB – MIMAROPA |  |  |  |
| Marinduque | 4 | 1 | 0 |
| Occidental Mindoro | 3 | 0 | 0 |
| Oriental Mindoro | 7 | 1 | 1 |
| Palawan | 3 | 1 | 0 |
| Romblon | 1 | 0 | 0 |
| Region V – Bicol Region |  |  |  |
| Albay | 9 | 0 | 0 |
| Camarines Norte | 9 | 5 | 1 |
| Camarines Sur | 96 | 54 | 7 |
| Catanduanes | 2 | 0 | 0 |
| Masbate | 10 | 2 | 0 |
| Sorsogon | 2 | 0 | 0 |
| Region VI – Western Visayas |  |  |  |
| Aklan | 3 | 2 | 1 |
| Antique | 0 | 0 | 0 |
| Capiz | 16 | 14 | 3 |
| Guimaras | 3 | 2 | 1 |
| Iloilo | 27 | 19 | 3 |
| Negros Occidental | 35 | 4 | 0 |
| Region VII – Central Visayas |  |  |  |
| Bohol | 2 | 1 | 1 |
| Cebu | 3 | 0 | 0 |
| Negros Oriental | 1 | 1 | 0 |
| Siquijor | 0 | 0 | 0 |
| Region VIII – Eastern Visayas |  |  |  |
| Biliran | 0 | 0 | 0 |
| Eastern Samar | 1 | 0 | 0 |
| Leyte | 6 | 0 | 0 |
| Northern Samar | 0 | 0 | 0 |
| Samar (Western Samar) | 3 | 2 | 1 |
| Southern Leyte | 1 | 0 | 0 |
| Region IX – Zamboanga Peninsula |  |  |  |
| Zamboanga del Norte | 2 | 0 | 0 |
| Zamboanga del Sur | 12 | 2 | 0 |
| Zamboanga Sibugay | 0 | 0 | 0 |
| Unknown (referred as "Zamboanga") | 3 | 3 | 0 |
| Region X – Northern Mindanao |  |  |  |
| Bukidnon | 12 | 1 | 0 |
| Camiguin | 3 | 1 | 1 |
| Lanao del Norte | 15 | 7 | 2 |
| Misamis Occidental | 1 | 0 | 0 |
| Misamis Oriental | 64 | 34 | 7 |
| Region XI – Davao Region |  |  |  |
| Compostela Valley | 2 | 0 | 0 |
| Davao del Norte | 3 | 0 | 0 |
| Davao del Sur (including Davao City) | 26 | 6 | 1 |
| Davao Oriental | 2 | 2 | 0 |
| Region XII – SOCCSKSARGEN |  |  |  |
| Cotabato City | 4 | 1 | 1 |
| Cotabato (North Cotabato) | 8 | 3 | 2 |
| Sarangani | 0 | 0 | 0 |
| South Cotabato (excluding General Santos City) | 1 | 1 | 0 |
| General Santos City (Dadiangas) | 2 | 0 | 0 |
| Sultan Kudarat | 3 | 1 | 0 |
| Autonomous Region of Muslim Mindanao |  |  |  |
| Basilan (excluding City of Isabela) | 2 | 0 | 0 |
| Lanao del Sur | 2 | 1 | 1 |
| Maguindanao (excluding Cotabato City) | 3 | 0 | 0 |
| Sulu | 0 | 0 | 0 |
| Tawi-Tawi | 1 | 1 | 0 |
| CARAGA |  |  |  |
| Agusan del Norte | 2 | 3 | 0 |
| Agusan del Sur | 5 | 1 | 0 |
| Dinagat Islands | 0 | 0 | 0 |
| Surigao del Norte | 0 | 0 | 0 |
| Surigao del Sur | 1 | 0 | 0 |
| Surigao City | 1 | 0 | 0 |
| TOTAL | 1032 | 497 | 73 |
